# The “*Journal of Functional Morphology and Kinesiology*” Journal Club Series: Highlights on Recent Papers in Athletic Training

**DOI:** 10.3390/jfmk3040049

**Published:** 2018-10-15

**Authors:** Silvio Lorenzetti, Antonino Bianco, Laura Stefani

**Affiliations:** 1 Swiss Federal Institute of Sport Magglingen, Hauptstrasse 247, 2532 Magglingen, Switzerland; 2 Sport and Exercise Sciences Research Unit, University of Palermo, 90100 Palermo, Italy; 3 Clinical and Experimental Medicine Department, School of Sports Medicine, Sports Medicine and Exercise Center, University of Florence, 50141 Florence, Italy

## Abstract

We are glad to introduce the tenth Journal Club. This edition is focused on several relevant studies published in the last years in the field of athletic training, chosen by our Editorial Board members and their colleagues. We hope to stimulate your curiosity in this field and to share with you the passion for the sport seen also from the scientific point of view. The Editorial Board members wish you an inspiring lecture.

## 1. Introduction

Even in Roman times, the gladiators knew that athletic training enhances muscle force, strength, and power [1]. Nowadays it is well known and accepted that an athletic training enhances the quality of life, performance, and general fitness. In order to improve cardiorespiratory and muscular fitness, improve bone health, and reduce the risk of NCDs and depression, the WHO [2] recommends moderate-intensity aerobic activity and strength training (for ages 18–64 years). In addition, for ages above 65 years, a training to enhance balance is recommended. A recent pooled analysis of 11 population cohorts, with a total of about 80,000 adults, clearly showed the influence on the mortality risk of different types of athletic training [3]. Here, interestingly, strength training resulted in a reduction of 23% in all-causes mortality and in a 31% reduction in cancer mortality. Remarkably, the cancer mortality was not reduced for aerobic training only. This clearly shows that not only exercising, but a wise combination of the different types of training is important.

In this editorial, several aspects of athletic training are covered. First, the technical assessment of training: here, the general trend of digitalization, with mobile sensors and smart watches, allows the quantification of training on a personal level. Then, the question about the importance of muscular strength for athletic training is elaborated. Furthermore, the importance of exercise in renal transplant recipients is debated from a sports medicine physician’s point of view.

## 2. Recent Papers Regarding Athletic Training

### 2.1. Technical Assessment of Athletic Training

#### Highlight by Silvio Lorenzetti

Nowadays, it is common to quantify athletic training [4]. The resulting data can be used to monitor the workout, to estimate the change in the performance, or to provide feedback to the athlete [5]. More mobile and relatively low-cost measurement devices for the personal athletic level are available, mainly in a nonlaboratory setting. These devices are more common in the area of aerobic training for analyzing the heart parameters, position, and velocity (via geographic positioning system; GPS), but have recently become more popular for strength training. Here, they measure the path of the barbell, count the repetitions, estimate the type of exercise, and determine the one repetition maximum and muscle fatigue. Recently, for each application, a specific sensor has been developed. For the future of most applications, it might be possible to use commercially available smart watches and focus on the development of algorithms to analyze the measured data, with a focus on the software side. Already, the first steps toward assessing stress levels using these devices have been completed [6]. There is no doubt that many more parameters will be assessed using these devices in the near future. 

In addition to the new technological possibilities and solutions, there are two crucial points to be considered. First, for all application types, proper validation and testing for reliability is required [7] in order to identify possibilities and limitations. The outcomes of these devices are often less accurate; therefore, the interpretation of the data should be completed with care [8]. Finally, the ethical component cannot be neglected. The personal data belongs to the athlete. The wearer or user needs to be able do decide who has access to this data. Questions about where is the data stored, how can it be accessed safely, how it can be deleted, and if the data can be shared in an anonymous manner must be clarified and addressed.

### 2.2. How Important Is Muscular Strength for Athletic Training

#### Highlight by Antonino Bianco

Muscular strength is the key determinant of any athletic performance and also can be considered as the central matter of interest during physical preparation periods and athletic training. The main paper for this commentary is the remarkable review published in 2016 by Suchomel et al., in which the authors highlighted how essential muscular strength is for human performance and then defined several key points that deserve attention and a deeper understanding [9]. The authors concluded that professionals and practitioners can manipulate absolute and relative muscular strength with regular training and this can provide to the athlete the capacity to improve abilities such as jumping, sprinting, and other sport-specific performances. Of interest, a remarkable role of muscular strength on decreasing injury rate also seems to be confirmed [9]. I strongly advise reading this manuscript and the following update (Suchomel et al. [10]) to have a clear picture of the matter according to the authors’ findings. 

The typology of strength required for sports performance may vary depending on the sports characteristics, and these relations have been well documented in the literature [11,12,13,14]. Notwithstanding the two papers mentioned above, which seem to be exhaustive and welcomed by the scientific community, not all concerns highlighted previously by McGuigan et al. in 2012 [11] have been addressed by the recent literature. In 2012, the authors in their brief review pointed out the difficulties of finding data coming from elite athletes. They also emphasized the fact that with the higher the level of the athlete, the higher the level of strength program specialization must be [11]; this appears, of course, reasonable, but it is helpful to be reminded of the fact that not all exercises are created equal [14], or as they stated, “not all training programs are created equal” [11]. 

Up to now, all papers have mentioned highlighting the importance of a minimum/adequate level of strength before starting with a specific power training oriented to the discipline. The serious debate is always the same: how much time and attention fitness coaches must dedicate to a particular biomotor ability (such as maximal strength, upper body explosive strength, and so on) rather than administering a competition’s conditions and power training exercises in order to replicate the competition’s situations perfectly.

In my personal opinion, it is clear that less muscular strength determines less muscular power, and then, as a consequence, this negatively affects performance, so professionals must continuously monitor the athlete’s muscular strength levels with proper field tests that accurately reflect the performance of interest (both at an Olympic level and at a local level). How important is muscular strength for athletic training? It is a fundamental part of both individual sport and team sports.

### 2.3. Exercise in Renal Transplant Recipients: A Sports Medicine Physician Can Prescribe Physical Activity

#### Highlight by Laura Stefani

Physical activity is an important tool for cardiovascular disease prevention in the general population [15], and much data has been recently emerging to support this positive effect in different kinds of chronic forms of metabolic disease [16,17]. Solid organ transplant recipients are the new category of subjects at high cardiovascular risk and affected by multiple metabolic chronic diseases that can be considered among those involved in the exercise program. Especially for renal transplant recipients (RTR), regular physical activity represents the best method to return to a normal lifestyle after long time of sedentarism, to prolong the patients’ survival and to maintain the organ normal function. Despite the surgical treatment, the patients still suffer from a cardiovascular morbidity and mortality risk that is much higher than in the general population [18,19]. Many factors contribute to increased cardiovascular mortality: prolonged inactivity, long time of dialysis exposure, and hypertension and diabetes often being present before the surgical treatment. A possible association to other cardiometabolic risk factors such as smoke, alcohol, and overweightness strongly related to incorrect lifestyle is found. Despite the fact that official guidelines for the exercise indications for transplant recipients are not yet available, the suggestions for a correct lifestyle, including regular moderate mixed physical activity and adherence to the nutritional habits program, are now the first approach to reduce the cardiometabolic risk factors. In addition, in RTR, as well in other solid organ transplant recipients, the use of steroid and immunosuppressive therapy can contribute to their body weight gain that may accelerate atherosclerosis in these patients [20]. 

For a long time, the RTR have been considered a frail category of patients, and the physical activity has been avoid on the basis of the hypothesis that it could potentially harm them. On the contrary, many trials have supported the role especially of supervised physical activity [21]. More recently, the unsupervised exercise has also been proposed to overcome the difficulties for a constant adherence [22]. The study demonstrates that especially the myocardial function parameters evaluated by echocardiography exam maintain a normal range, with evidence of a progressive improvement of the contractility estimated by deformation parameters, as strain analysis shows [23]. A preliminary investigation of eventual cardiotoxicity is therefore important, especially in the case of a temporary absence of supervision on behalf of dedicated professional figures, such as “physiokinesiologists”. Some other parameters, such as upper and lower limbs’ power evaluation by hand-grip and chair tests, flexibility by the “Sit and Reach” test, ergometric tests or cardiometabolic tests, if possible, as well as the study of body composition (in terms of water distribution and fatty free mass/fatty mass ratio, which are fundamental to complete the check-up for providing data to the sports medicine specialist to plan the exercise program. 

In conclusion, sports medicine is the appropriate specialist context that, starting with the specific experience, can offer all the instruments for the correct management of RTR patients. 

The integration with other medical and nonmedical professionals adds more value to our daily work.
